# Evaluation of Potential Probiotic Properties and In Vivo Safety of Lactic Acid Bacteria and Yeast Strains Isolated from Traditional Home-Made Kefir

**DOI:** 10.3390/foods13071013

**Published:** 2024-03-26

**Authors:** Angela Maione, Marianna Imparato, Annalisa Buonanno, Maria Michela Salvatore, Federica Carraturo, Elisabetta de Alteriis, Marco Guida, Emilia Galdiero

**Affiliations:** 1 Department of Biology, University of Naples “Federico II”, 80126 Naples, Italy; angela.maione@unina.it (A.M.); marianna.imparato@unina.it (M.I.); annalisa.buonanno@unina.it (A.B.); federica.carraturo@unina.it (F.C.); dealteri@unina.it (E.d.A.); marco.guida@unina.it (M.G.); 2 Department of Chemical Sciences, University of Naples “Federico II”, 80126 Naples, Italy; mariamichela.salvatore@unina.it; 3 BAT Center-Interuniversity Center for Studies on Bioinspired Agro-Environmental Technology, University of Naples Federico II, 80055 Portici, Italy

**Keywords:** kefir grains, probiotics, lactic acid bacteria, *Pichia*, biofilm, cell adhesion, *Galleria mellonella*

## Abstract

Probiotics are known for their health-promoting resources and are considered as beneficial microorganisms. The current study focuses on the isolation, and on a complete in vitro and in vivo characterization, of yeast and lactic acid bacteria acquired from traditional homemade kefir in order to assess their potentiality as probiotic candidates. In particular, the isolates *Pichia kudriavzevii* Y1, *Lactococcus lactis* subsp. *hordniae* LAB1 and *Lactococcus lactis* subsp. *lactis* LAB2 were subjected to in vitro characterization to evaluate their suitability as probiotics. Resistance to acid and bile salts, auto-aggregation, co-aggregation, hydrophobicity, and biofilm production capability were examined, as well as their antioxidant activity. A safety assessment was also conducted to confirm the non-pathogenic nature of the isolates, with hemolysis assay and antibiotic resistance assessment. Moreover, mortality in the invertebrate model *Galleria mellonella* was evaluated. Current findings showed that *P. kudriavzevii* exhibited estimable probiotic properties, placing them as promising candidates for functional foods. Both lactic acid bacteria isolated in this work could be classified as potential probiotics with advantageous traits, including antimicrobial activity against enteric pathogens and good adhesion ability on intestinal cells. This study revealed that homemade kefir could be a beneficial origin of different probiotic microorganisms that may enhance health and wellness.

## 1. Introduction

Probiotics have been defined as “live microorganisms which when administered, in adequate amounts, confer a health benefit to the host” [1]. For this reason, probiotics play a very important role in preserving human health as complementary therapy, sometimes preventing the use of conventional drugs, thanks to their essential role in the maintenance of gut microbiota function and composition, in the prevention of pathogen growth and in the improvement of overall digestive system function. In order to be used as a probiotic and function adequately in exerting beneficial effects, a microbial strain must present various desirable characteristics, such as gastrointestinal tolerance and the ability to colonize the human host. Furthermore, the probiotic candidate should be safe for humans and appropriate for biomedical purposes, with antipathogenic capabilities. In fact, the antiinfective mechanisms of probiotics includes regulation of gut microbiota, promotion of intestinal barrier function, attenuation of liver disease, competition for adhesion and nutrition, modulation of the immune system of the host, synthesis of antimicrobial compounds, and anticancer potential [2]. As reported by Žuntar et al., nowadays probiotics are classified into three categories: Foods, with claimed GRAS (Generally Recognized As Safe) status for *Lactobacillus*, *Bifidobacterium*, and *Lactococcus*; dietary supplements; and pharmaceuticals. The categorization depends on demands from different regulatory authorities on probiotic manufacturers and indications of use [3]. In recent years, several beneficial probiotics have been isolated from the environment and from fermented foods [4]. Microbial fermentation is a well-established and widely adopted technology to preserve foods and increase their shelf-life, also preventing human illnesses.

Most probiotics are bacteria, mainly belonging to the genera *Lactobacillus* and *Bifidobacterium.* The lactic acid bacteria (LAB), such as *Lactobacillus*, *Lactococcus*, *Enterococcus*, and *Pediococcus,* are important for the balance of the gut microbiome, biotherapeutic applications, antimicrobial potential, and their immunomodulatory effects, which have to be considered in a potential probiotic, in order that it be safe when dispensed in controlled quantities. *L. lactis,* has been well studied and, thanks to the production of powerful bioactive molecules, peptides, proteins, and live cells, it is already used in the production of food and healthcare products [5]. Although *L. lactis* is effective as a probiotic, this property is strain-specific and not common to all *L. lactis* [6]. Therefore, it is important to examine the characteristics of each strain particularly before classifying isolates as potential probiotics. Nowadays, the potentiality of yeasts as probiotics, as opposed to bacteria, is less commonly explored. In fact, only few researchers have investigated the efficacy of probiotic yeasts, like *Saccharomyces boulardii* and *Kluyveromyces marxianus,* which are the only two commercialized yeasts available until now [7,8,9,10]. *S. boulardii* has been selected as a non-bacterial example of a probiotic with activity against various gastrointestinal disorders [11]. Furthermore, recent studies have demonstrated the existence of other yeast species with potential probiotic characteristics, such as *K. marxianus*, the predominant species present in kefir grains [12], and *P. kudriavzevii*, generally present in the environment and mostly associated with naturally fermented foods, such as fruit, cocoa, coffee beans, cheese and yogurt [13].

Isolation and identification of other yeast species or strains with potential probiotic properties would be of great interest for both the food and pharmaceutical industries.

Kefir is a product obtained from the fermentation of milk or sugar solutions that differs from other fermented products due to the specific property of its starter: the grain [14]. The microbiota composition of kefir grains is influenced by its geographical origin, the substrate (dairy or non-dairy) and fermentation conditions, such as fermentation time, temperature, degree of agitation and the ratio of kefir grains to substrate [15,16]. Several studies have suggested that beneficial properties can be connected with a habitual consumption of kefir. For instance, statistical data show that people who include kefir in their diet are more longevous than those who do not [16]. Kefir has a long list of reported health benefits, including antimicrobial, hypo-cholesterolemic and hypo-tensive effects, along with its role in controlling glycemic control, and its antioxidant, anticancer, antiallergic, immuno-modulatory and antiinflammatory properties. In addition, its regular consumption is associated with improved lactose digestion and tolerance [17] so that it can become a kind of functional food [3,16]. The broad range of beneficial effects of kefir can be associated with the probiotic properties of microorganisms present in it, which can influence health directly or indirectly through their metabolites, improving, among other factors, behavioral disorders, stress and anxiety [18] and having a possible positive impact on atopic dermatitis [19]. It is also necessary to evaluate and screen safe starter cultures to enhance the safety and quality of kefir [20].

The aim of the present study was to isolate new LAB and yeast strains from an unexplored home-made traditional kefir and evaluate their potentiality for probiotic use. Indeed, isolates from a homemade kefir may differ in their features from strains generally present in commercially available products.

Therefore, characteristics of the isolates such as tolerance to gastric and bile acids, auto- and co-aggregation abilities, hydrophobicity, antioxidant power, biofilm formation, adhesion to intestinal cell lines, and antagonistic activity against some pathogenic intestinal bacteria have been determined. Furthermore, safety of the isolates was assessed both in vitro, evaluating sensitivity to antimicrobials and hemolysis, and in vivo, with the assessment of their survival rate in the model organism *Galleria mellonella.* According to the results obtained, further studies would be of interest for the possible use of these strains as candidates for probiotic use.

## 2. Materials and Methods

### 2.1. Kefir Grains

Homemade kefir, prepared by fermentation at room temperature, of commercial UHT (ultra-high temperature pasteurized) semi-skimmed cow milk was provided by a private Italian family.

In the laboratory, kefir grains were propagated in 10% *w*/*v* sterile reconstituted skimmed milk (Sigma Aldrich, St. Louis, MO, USA) at room temperature for 24 h, simulating homemade preparation, and stored at 4 °C (for short-term storage) or at −80 °C (for long-term storage).

### 2.2. Isolation of Yeasts and LABs from Kefir Grains

In order to isolate microorganisms, 10 g of kefir grains sample were suspended in 90 mL of PBS and homogenized with Fisherbrand 850 Homogenizer (Thermo Fisher Scientific, Waltham, MA, USA) for 20 min. The suspended sample was serially diluted in PBS and plated onto Rosa Bengal agar (RB, Sigma-Aldrich, St. Louis, MO, USA), supplemented with chloramphenicol to isolate yeast and onto Man Rogosa and Sharpe Agar (MRS, Oxoid Ltd., Basingstoke, UK) to isolate LAB. The plates were incubated at 25 °C for 96 h and at 37 °C for 48 h at aerobic conditions, respectively [21,22]. Subsequently, the colonies differing in morphology were isolated for molecular identification.

### 2.3. Molecular Characterization of Selected Isolates

#### Bacterial and Fungal DNA Extraction, Amplification and Sequencing

Bacterial strains were sub-cultured on Tryptone Soya Agar (TSA, Thermo Fisher Scientific Inc., Waltham, MA, USA). Isolated colonies, homogenized in 70 μL Milli-Q Type 1 Ultrapure Water, underwent DNA extraction through denaturation for 10 min at 98 °C. Samples were centrifuged for 5 min at 8000 rpm at 4 °C and supernatant was subsequently recovered and employed for amplification through PCR; reactions were performed in a TECHNE Prime Thermal Cycler using the V3_f-V6_r universal primer set, targeting 16 S rRNA gene’s V3 and V6 regions of (5′-CCAGACTCCTACGGGAGGCAG-3′ and 5′-TCGATGCAACGCGAAGAA-3′; 700 bp amplicon size). Reactions were carried out according to Carraturo et al., in 200 µL sterile vials using: 5.5 µL PCR Key Buffer Tripton Free (Tris-HCl pH 8.5, KCl, 15 mM McCl_2_), 1.0 µL dNTP 12 µM, 0.22 µL forward primer and 0.22 µL reverse primer both 50 µM, 0.5 µL Taq polymerase, 1.3 µL DNA and 47 µL sterile deionized water, reaching a 55 µL final volume, and the following incubation conditions: initial denaturation at 95 °C for 2 min; 35 cycles of 95 °C for 30 s, 62 °C for 30 s; 72 °C for 30 s; a final extension of 72 °C for 5 min [23]. Fungal strain was sub-cultured on Dichloran Rose Bengal Chloramphenicol agar (DRBC, Thermo Fisher Scientific Inc., Waltham, MA, USA). CTAB extraction protocol [24] was employed to extract fungal DNA. Extracted DNA were amplified with PCR, targeting ITS-5.8S rDNA region of the fungal 18S rRNA gene (750 bp amplicon size) [25], employing a TECHNE Prime Thermal Cycler, and disposing of a ITS1_f (5′-GGA AGT AAA AGT CGT AAC AAG G-3′ 5′-TCC GTA GGT GAA CCT GCG G-3′) and ITS4_r (5′-TCC TCC GCT TAT TGA TAT GC-3′) primer set (Biofab Research, Rome, Italy). Templates were run on a 1.5% agarose gel, stained with GelRed (BIOTIUM), utilising a 100 bp DNA ladder as a reference. Sanger Sequencing reactions were performed by an external service (Biofab Research, Rome, Italy); the obtained FASTA sequences were interpreted using an editing tool, Chromas Lite v. 2.6.6 (Technelysium Pty Ltd., South Brisbane, Australia), and compared to NCBI Sequence Database sequences, using BLASTN ver. 2.2.29 (also referring to GenBank), selecting the highest percentage Identity, with a 98% cut-off and 0.0 e-value.

### 2.4. Probiotic Evaluation

#### 2.4.1. Acid and Bile Salt Tolerance

Acid and bile resistances were determined as reported previously [26]. Briefly, each isolate (Y1, LAB1 and LAB2) was collected from a18 h culture in YPD for Y1 or MRS for LAB strains at 37 °C. The cell suspensions were washed with PBS and resuspended in 5 mL PBS plus HCl (pH = 3.0) or PBS plus 0.3% *w*/*v* bile salts (Sigma Aldrich St. Louis, MO, USA), so as to have an initial cell density of 10^8^–10^9^ cells/mL. The suspensions were incubated for 4 h at 37 °C, in an orbital incubator at 150 rpm. The number of residual viable cells was counted every hour using the standard plate count method.

#### 2.4.2. Auto-Aggregation/Co-Aggregation Capacity

To determine auto-aggregation [27], overnight microbial cultures of the three strains (Y1, LAB1, LAB2) were centrifuged for 10 min at 5000× *g* at 4 °C, washed twice, resuspended and adjusted to OD_590_ = 0.6–0.8 in 5 mL of phosphate buffered saline (PBS), then incubated for 2, 4 and 24 h at 37 °C. An aliquot of the suspension was delicately removed from the upper zone and OD_590_ was measured before (OD0) and after (ODt) incubation. The auto-aggregation (A) percentage was calculated as:

A=[1−(ODt/ODt0)]×100.

For the co-aggregation assay, 4 mL of each strain and 4 mL of each pathogen culture (*E. coli* ATCC 25922, *L. monocytogenes* ATCC 7644, *Salmonella* spp. called S1, S2, S3, S4) were mixed, vortexed for 10 s, and incubated for 2 and 4 h at 37 °C. Each control tube contained 4 mL of each single suspension. The absorbance (OD) of each mixed suspension was then measured at 590 nm (ODmix) and compared with those of the control tubes containing the yeast and LAB strains (ODstrain) and the specific pathogen (ODpathogen) after incubation. The % of co-aggregation was calculated as

C=[1−ODmix×(ODstrain+ODpathogen)/2]×100.

#### 2.4.3. Hydrophobicity Assays

Cell biomass was suspended in 5 mL of PBS and absorbance at 600 nm was measured (OD0). Then, 3 mL xylene, were added to each sample. After incubation at 37 °C for 60 min without shaking, the absorbance of the interphase was measured (ODF) [28]. The hydrophobicity percentage was calculated as follows:

H=[1−(ODF/OD0)]×100%

#### 2.4.4. Adhesion to Caco-2 Cells and HT29 Cells

Human Caucasian colon adenocarcinoma Caco-2 cells and human colon epithelial mucus-secreting HT29 cells (ATCC collection) were both cultured in Dulbecco’s modified Eagle’s medium (DMEM; Sigma Aldrich Co., St. Louis, MO, USA), supplemented with 10% *w*/*v* fetal bovine serum (FBS; Sigma Aldrich Co., St. Louis, MO, USA), 2 mM L-glutamine (Sigma Aldrich Co., St. Louis, MO, USA), and 1% *w*/*v* penicillin–streptomycin (Sigma Aldrich Co., St. Louis, MO, USA) at 37 °C, in a 5% CO_2_ atmosphere. Cells were inoculated at final concentration of 2.5 × 10^5^ cells/well in 12-well plates containing DMEM without penicillin–streptomycin and incubated for 24 h at 37 °C, 5% CO_2_. Following the incubation period, Y1 at a concentration of 10^6^ and the two LAB strains at a concentration of 10^8^ were added to the human cells monolayer and incubated for 2 h in the same conditions. Then, wells were washed with PBS, and microbial-adhered cells were recovered by scraping. Serial dilutions were made in PBS and 100 µL were plated onto chloramphenicol RB agar or onto MRS agar for Y1 or the two LAB strains, respectively. The plates were incubated at 37 °C for 24–48 h, then, the CFUs were counted and the results were expressed as the mean of Log_10_ CFU/mL [29]. The percentage of microorganisms adhered to human cells was calculated as follows:

Adhesion(%)=Log10(CFU/mL) adhered microorganisms/Log10(CFU/mL) added microorganisms ×100

#### 2.4.5. Quantitative Assessment of Biofilm Formation

This was performed as described previously by Maione et al. [30] and detected using the crystal violet staining method for total biofilm biomass determination, according to Stepanovi’c et al. [31].

In short, 100 mL of each individual culture or of the mix of the three microorganisms together at a final concentration of 1 × 10^6^ CFU/mL was pipetted into wells of a polystyrene 96-well microplate. After 24 h at 37 °C, the microplate was rinsed three times with PBS, fixed with 99% methanol for 15 min, and air-dried before adding 200 μL of 1% *w*/*v* crystal violet (CV) (Sigma-Aldrich, St. Louis, MO, USA) to each well in order to detect the biofilm forming cells.

Optical density of stained adherent cells was determined at 570 nm with an automatic microplate reader (SYNERGY H4 BioTek, BioTek Instruments, Agilent Technologies, Winooski, VT 05404, USA). Experiments for each strain were performed in triplicate. The cut-off OD (ODC) was defined as three standard deviations above the mean OD of the negative control. The ability to form biofilm was determined as follows: OD ≤ ODC as non-biofilm producer, ODC < OD ≤ 2ODC as weak biofilm producer, 2ODC < OD ≤ 4ODC as moderate biofilm producer, and OD > 4ODC as strong biofilm producer. To determine the viable microbial cells in the biofilm, the plate count method was used. Biofilm was washed three times to eliminate non-adherent cells, detached and resuspended in PBS. 100 µL of the biofilm suspension was plated onto RB (Sigma-Aldrich, St. Louis, MO, USA), supplemented with chloramphenicol for the yeast and onto MRS agar supplemented with plus 10 μg/mL Amph B for the LAB strains. The plates were incubated at 37 °C for 24–48 h, after which the CFUs were counted.

#### 2.4.6. Preparation of Cell-Free Supernatant (CFS) and Antimicrobial Activity against Food-Borne Pathogens

Each isolate alone or all three together were grown in MRS/YPD broth for 48 h at 37 °C. Cell free supernatants (CFSs) were collected by centrifugation for 30 min at 4000× *g*, 4 °C, and were adjusted to pH 7 using 1 M of NaOH to eliminate the organic acids’ effects and then filter-sterilized through a 0.22 μm pore filter. Overnight cultures of three *Salmonella* spp (S1, S2, S3, S4) obtained from food isolation and kept in our laboratory Culture Collection, *E. coli* ATCC 25,922 and *L. monocytogenes* ATCC 7644 were spread onto nutrient agar (NA, Sigma-Aldrich, St. Louis, MO, USA) plates and wells were made on it. The CFS (25 μL/well) was dispensed into the well which was made on NA and incubated at 24 h for 37 °C. The inhibitory activity was monitored and compared [32]. Diameters of circular inhibition zones produced around the CFSs were measured in mm [33]. Screening and identification of probiotic Lactobacilli from the infant gut microbiota was carried out to mitigate mean toxicity [33]. The test was repeated twice for each strain.

#### 2.4.7. Antioxidant Activity

The DPPH antioxidant activity of Y1, LAB1 and LAB2. 466 strains was evaluated with minor modifications according to the method of Merchan et al. [34]. In brief, 800 μL of each strain suspension was mixed with 1 mL of 0.2 mmol/L 1,1-diphenyl- 2-picrylhydrazyl solution in methanol. The samples were incubated for 30 min in the dark at room temperature with agitation and then centrifuged at 2000 rpm for 2 min. The supernatant absorbance was measured at 517 nm. The scavenging ability (SA) was calculated using the formula:

SA=[1−OD517 (sample)/OD517 (blank)]×100

### 2.5. Safety Assessment

#### 2.5.1. Hemolytic Activity

The hemolytic activity of the three isolates was carried out according to the method described by Shen et al. [35] by inoculating the strains on blood agar plates (Oxoid Ltd., Basingstoke, UK) containing 5% defibrinated sheep blood for 48 h of incubation at 37 °C in order to detect patterns of hemolysis. Nex, cultured plates were observed to assess a hemolytic zone, and a clear zone of hydrolysis around the colonies was considered as a positive result (β-hemolysis).

#### 2.5.2. Antibiotic and Antifungal Assistance

Antimicrobial resistance was screened by agar disk diffusion method as described by Turchi et al. [36] in accordance with the Clinical and Laboratory Standards (M44, M45). The isolated yeast strain was tested for resistance against the most commonly used antibacterial and antifungal comp0unds, 12 belonging to the first category and 5 to the latter, each of which has a different mode of action. The molecules tested were gentamicin (10 μg GM), ampicillin (10 μg AMP), polymyxin-B (300 U PB), amoxycillin + clavulanic acid (30 μg AMC), norfloxacin (10 μg NOR), streptomycin (10 μg STR), cephotaxime (30 μg CTX), ciprofloxacin (10 μg CIP), azithromycin (15 μg AZ), ceftriaxone (30 μg CRO), vancomycin (20 µg VAN), rifampicin (20µg RIF), fluconazole (10µg FL) caspofungin (2 µg CSF), ketoconazole (30 µg KET), itraconazole (30 µg ITR), and amphotericin B (2 µg Amph B). After 24–48 h incubation at 30 °C, the diameter of the inhibition zone was measured and the strains were considered susceptible (S), intermediate (I), or resistant (R).

### 2.6. In Vivo Safety Analysis

#### 2.6.1. *Galleria mellonella* Survival Assay

Larvae of *G. mellonella* were used to evaluate the safety of the three strains and CFS, performed as previously reported [37] with a slight modification. For each assay, 20 healthy larvae, randomly chosen and of a similar size, were selected and injected with 10 μL of cell yeast/bacteria suspension through the last left pro-leg, with a final inoculum concentration in PBS buffer of 10^7^, 10^6^, 10^5^, 10^4^ cells/larva or with CFS-Y1, CFS-LAB1, CFS-LAB2 and CFS-Mix, and kept at 37 °C. Sterile PBS alone or intact larvae were used as controls. Every 24 h throughout the 72 h follow-up, the number of viable larvae was registered, and the percentage survival was calculated. Larvae were considered dead when they displayed no response to touch.

#### 2.6.2. Burden Assay

The fungal/bacterial burden in larvae was assessed after 24 h of inoculation for the three doses of treatment, 10^4^, 10^5^, 10^6^, and 10^7^ CFU/larva, as reported previously [38]. Three survival larvae from each treatment group were homogenized in sterile PBS by vortexing and 100 μL of each sample was collected and serially diluted and plated onto YPD agar containing 20 μg/mL chloramphenicol (Oxoid, UK) to inhibit bacterial growth, or MRS plus 10 μg/mL Amph B to inhibit yeast growth, respectively. The experiments were performed in triplicate. Plates were incubated at 30 °C for 24–48 h, and the cell concentration (CFU/mL) was calculated for each treatment.

### 2.7. Statistical Analyses

GraphPad Prism Software (version 8.02 for Windows, GraphPad Software, La Jolla, CA, USA, www.graphpad.com, accessed on 14 February 2024) was used for data analysis. All data are shown as mean ± standard deviation (SD) and were derived from two or three independent experiments. One-way or two-way analysis of variance (ANOVA) followed by Tukey’s test was used for the comparison test. The Kaplan–Meier method and log-rank (Mantel–Cox) test were used to plot survival curves.

## 3. Results

### 3.1. Isolation and Molecular Characterization of Yeast and LABs from Kefir Grains

Phenotypic characterization of the microbial isolates from kefir grains, regarding colony morphology, revealed that all isolates on MRS agar had white or creamy colonies and corresponded to Gram-positive bacteria, while colonies on RB agar corresponded to yeast colonies, as observed microscopically.

However, to assign reliable profiles to the selected isolates, molecular identification remains essential and obligatory. Results of Sanger Sequencing of isolated bacterial and fungal colonies are reported in Table 1.

Three isolates were identified and corresponded to *P. kudriavzevii* (Y1), *L. lactis* subsp. *hordniae* (LAB1) and *L. lactis* subsp. *lactis* (LAB2).

### 3.2. Survival under Simulated Gastrointestinal Tract Conditions

**Figure 1:**
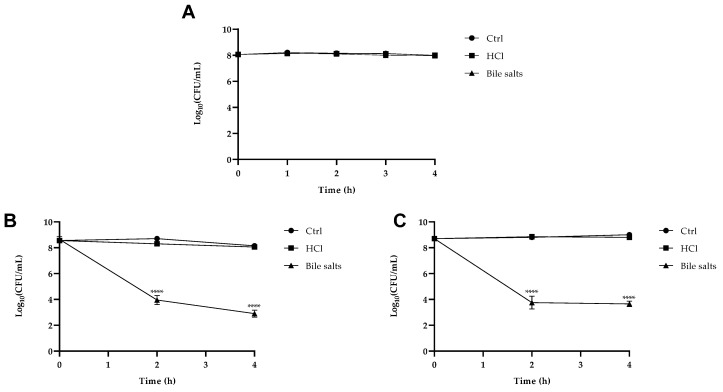
Cells viability of *P. kudriavzevii* (**A**), *Lactococcus lactis* subsp. *hordniae* (**B**) and *L. lactis* subsp. *lactis* (**C**) when submitted to simulated gastric transit. Asterisks represent significant differences vs. control (Ctrl), **** *p* < 0.0001.

To examine whether Y1, LAB1 and LAB2 were able to reach the intestine alive, we examined (Figure 1A–C) their survival under various conditions (presence of HCl and bile salts). As reported in Figure 1A, the *P. kudriavzevii* showed tolerance to both HCl and bile salts until 4 h, as no difference with the control was observed, proving that the isolated yeast strain can survive in both stomach and intestine without becoming degraded during digestion through the gastrointestinal tract. Instead, as shown in B and C, both the two LABs in this experiment survived at pH 3, but were affected by bile salt treatment, since the number of viable bacteria decreased by about 4 Log within 4 h (*p* < 0.0001).

### 3.3. Auto-Aggregation and Hydrophobicity Assay

**Figure 2:**
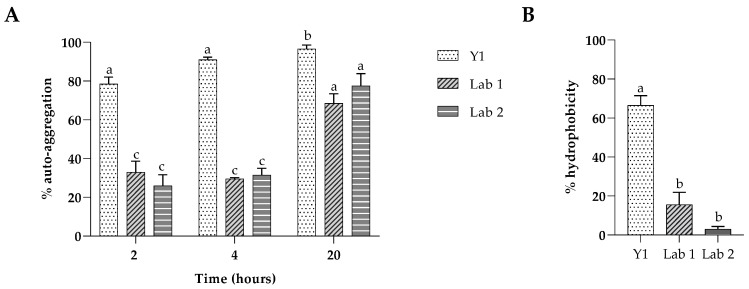
(**A**) Percentage of auto-aggregation of *P. kudriavzevii* (Y1), *Lactococcus lactis* subsp. *hordniae* V4048 (LAB 1) and *L. lactis* subsp. *lactis* NM26-6 (LAB 2); (**B**) percentage of hydrophobicity of *P. kudriavzevii* (Y1), *L. lactis* CM-CNRG466 (LAB 1) and *L. lactis* subsp. *lactis* NM26-6 (LAB 2). ^a, b, c^ Significantly different (*p* < 0.05).

The results in Figure 2A show that the auto-aggregation percentages varied among the tested strains. The highest value of about 80% was detected for *P. kudriavzevii* after 2 h, 90% after 4 h, reaching about 100% after 20 h incubation. The result was consistent with those reported in previous studies where auto-aggregation ability of yeast strains increased throughout 24 h and, especially for *Pichia kudriavzevii*, the highest percentage of auto-aggregation (95%) was detected after 20 h [39]. The LAB strains showed intermediate auto-aggregation ability (Figure 1A) with mean values between 30% and 35%, reaching 75–80% after 20 h incubation. The hydrophobic property of the cell surface is considered another important factor in the adhesion and proliferation of microorganisms on intestinal epithelial cells determining their colonization capacity, which is a crucial step in the proliferation of probiotics in the intestine.

Variability was observed in our strains for all three isolates (Figure 2B), with hydrophobicity values for *P. kudriavzevii* above 50%, reaching 65% after 1 h of incubation. Instead, LAB1 and LAB2 revealed negative results, reaching less than 20% hydrophobicity.

### 3.4. Co-Aggregative Effect of Y1, LAB1 and LAB2 against Intestinal Bacteria

The ability to co-aggregate can prevent pathogenic bacteria, which can adhere to intestinal cells, from colonizing the intestine. The results (Table 2) show that the co-aggregation values were higher after 4 h, compared to those after 2 h of incubation. *P. kudriavzevii* showed the best percentage of co-aggregation after 4 h against S3, *L. monocytogenes* and S4, at 82.6%, 81.6% and 79.8% respectively (*p* < 0.005). Lower co-aggregation values were found for the two LAB strains with the lowest percentage of 21.3 and 20.7 against S4.

### 3.5. Adhesion Capacity

**Figure 3:**
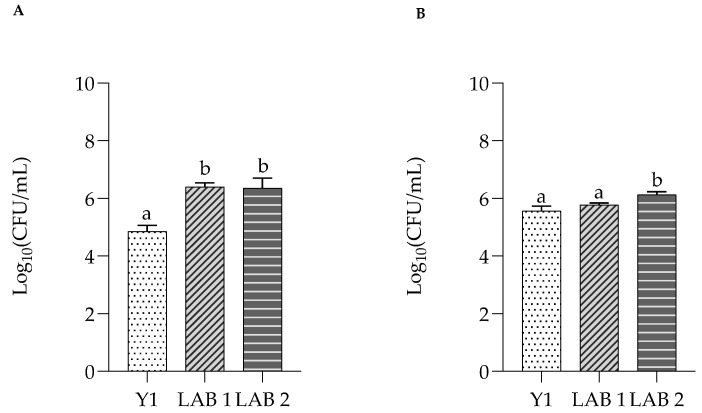
Adhesion capacity of Y1, LAB1, and LAB2 to Caco-2 (**A**) and HT-29 cells (**B**). ^a, b^ Significantly different (*p* < 0.05).

Adhesion to intestinal epithelial cells is an important requisite for the colonization of probiotic strains in the intestine, establishing a physical barrier against the adhesion of pathogens [40]. Y1, LAB1 and LAB2 isolated from kefir grains adhered to Caco-2 cells or HT-29 cells in 2 h and are depicted in Figure 3A,B. Y1 reached a cell density of 4.85 Log CFU/mL and 5.78 Log CFU/mL on Caco-2/HT-29 cells, corresponding to 80.83% and 93% adhesion, respectively, values higher than those reported in other studies [41].

LAB1 and LAB2 reached a cell density of 6.40 and 6.35 Log CFU/mL, corresponding to values of adhesion of 80 and 79.3%, respectively, on CaCo-2 cells. On HT-29 cells, the values were 5.78 and 6.12 Log CFU/mL, corresponding to 72% and 76.5% for LAB1 and LAB2, respectively, indicating a performing adhesion ability with intestinal cells. Consistent with the results of the present study, previous studies revealed that percentages of adhesion to intestinal cell mono-layers were similar, and in agreement with values obtained for other kefir derived microorganisms for Caco-2 and HT-29 cells [42].

### 3.6. Determination of the Optimal Biofilm Formation

Biofilm is a consortium of microorganisms in which cells attach to each other and to a surface, and are surrounded by an extracellular matrix (EPS) produced by the microorganisms themselves [43]. In this study, the three isolates were able to form biofilm. Y1 had good biofilm formation ability, as shown in Table 3, and this result was in agreement with its good auto-aggregation capacity and hydrophobicity, which indicates not only that the hydrophobic and non-polar surface of the microorganisms facilitated cell adhesion by forming a protective barrier in the human intestine, but also favoring biofilm formation. Conversely, the two LAB strains showed a weaker capacity to form biofilms on polystyrene after 24 h (Table 3). Interestingly, the co-cultivation of yeast with each LAB strain promoted a better biofilm formation ability that further increased when the three strains were co-cultured. For all three isolates, the number of adherent viable cells (CFU/mL) in the biofilm was reported (Table 3).

### 3.7. Activity of CFSs against Some Food-Borne Pathogens

CFSs were collected after 48 h from the isolates, grown alone or all three mixed.

Zone inhibition observed (Table 4), indicated that the supernatant of the *P. kudriavzevii* did not show any antimicrobial activity against the microorganisms tested. Instead CFSs from LAB1 and LAB2 cultures showed a high level of antimicrobial activity with a zone of inhibition measurements of 11.5 and 12.6 mm for *L. monocytogenes* respectively and 11 and 11.9 mm for S4 respectively confirming an antagonist activity and consequently the possibility to be used in food and in pharmaceuticals. No activity was observed against *E. coli* ATCC 25922 and S2. Likewise, a positive effect with a moderate inhibitory activity was observed for the CFS-MIX in reducing the colonization.

Presumably these results could be attributed to the production from the LAB strains of some metabolites like bacteriocins with antimicrobial properties, in agreement with numerous findings [11,44], where the inhibitory properties are considered a relevant beneficial probiotic property of LAB strains for food preservation and prevention of food-borne pathogens.

### 3.8. Antioxidant Activity

**Figure 4:**
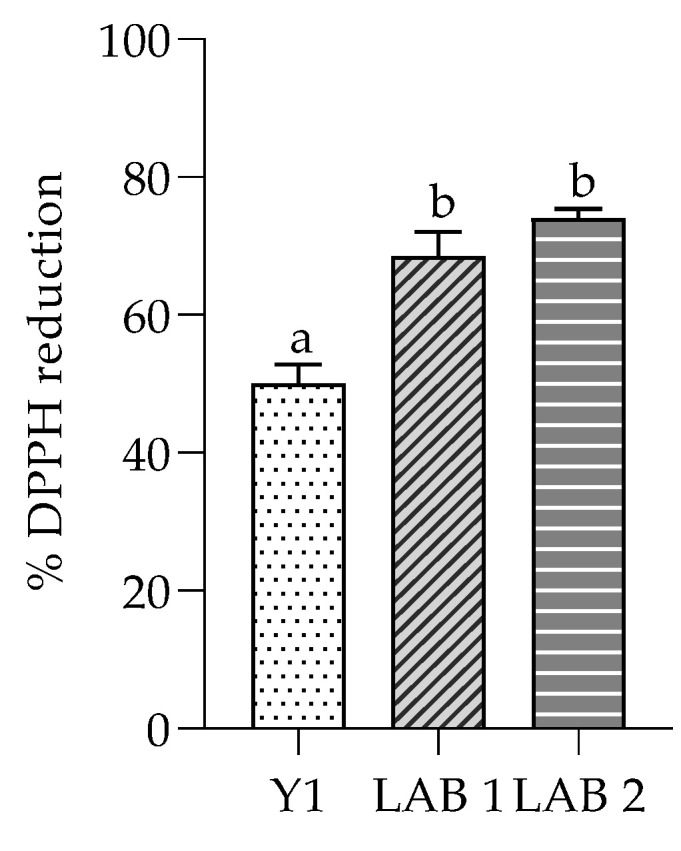
Antioxidant activity of Y1, LAB 1 and LAB 2 on DPPH. ^a, b^ Significantly different (*p* < 0.05).

The isolates displayed distinct antioxidant activities, as evaluated by the DPPH method (Figure 4). The antioxidant activity presented by Y1 was about 50%, in line with studies conducted by other researchers that found a DPPH scavenging capacity ranging from 48 to 64% [34,45]. Our results were consistent with previous research [46], showing an excellent antioxidant activity also for LAB. Indeed, LAB1 and LAB2 had a high scavenging capacity, at 70 and 80%, respectively, such as to make them two potential probiotic strains.

### 3.9. Safety Assessment

**Figure 5:**
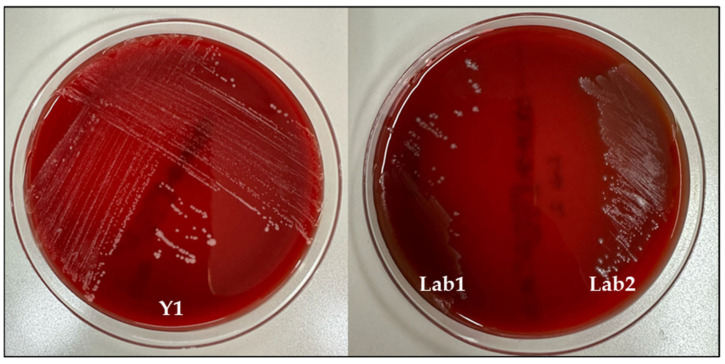
Hemolytic activity of Y1, LAB 1 and LAB 2.

The safety of these strains was assessed through the performance of two tests: hemolytic activity and antifungal/antibacterial drug resistance. As shown in Figure 5, no hemolysis was observed when these isolates were streaked on Columbia 5% sheep blood agar plates, which validates the safety of the isolates. Both antifungal and antibacterial drugs were used in the experiment. In accordance with the CLSI M45 guidelines, both *L. lactis* LAB 1 and LAB 2 (Table 5) were susceptible to the antibiotics tested, showing a clear inhibition diameter. As reported in the same table, the yeast isolate was resistant to antibiotics and sensitive to different antifungal agents, except for ITR. Antibiotic sensitivity was tested to demonstrate the resistance of *P. kudriavzevii,* in order to make it suitable for probiotic formulation, as being capable of maintaining the intestinal microbiota without being affected by antibiotics prescribed during the pharmacological therapies. The resistance towards all the tested antibiotics confirms that *P. kudriavzevii* from our kefir could be prescribed to patients under antibiotic treatment. Antimycotic sensitivity, on the contrary, was tested to show the possibility of their use in yeast infection.

### 3.10. In Vivo Safety Analysis

**Figure 6:**
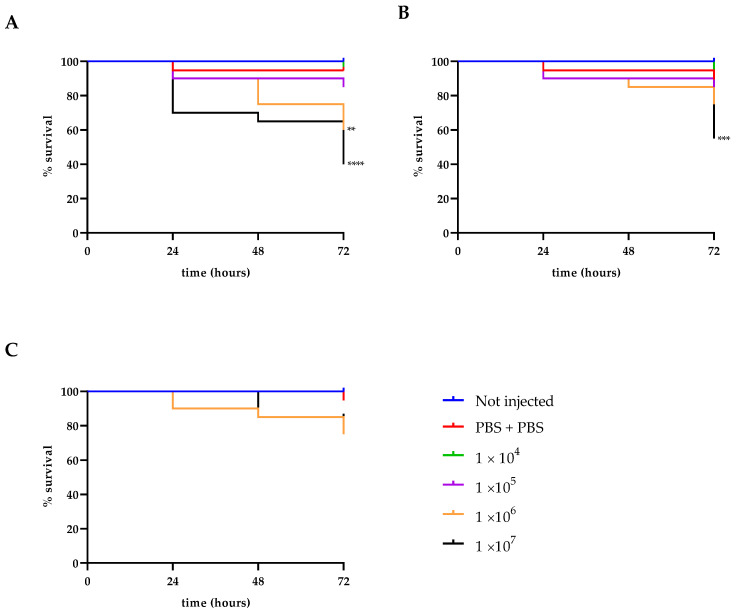
Survival curves of *G. mellonella* larvae injected with *P. kudriavzevii* (**A**), *Lactococcus lactis* subsp. *hordniae* (**B**) and *L. lactis* subsp. *lactis* (**C**) at concentrations ranging from 1 × 10^4^ to 1 × 10^7^ CFU/larvae. Asterisks represent significant differences vs. control (Not injected), **** *p* < 0.0001, *** *p* < 0.001, ** *p* < 0.0001.

**Figure 7:**
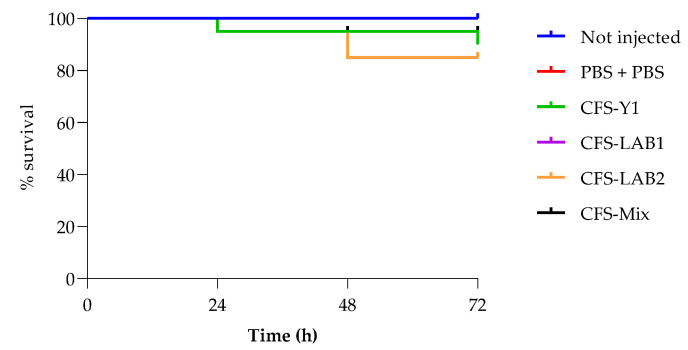
Survival curves of *G. mellonella* larvae injected with CFS of Y1, LAB 1, LAB 2 and CFS of all the three strains together (CFS-MIX).

To assess the virulence of these strains in the insect larvae *G. mellonella*, the survival rate in larvae challenged with an inoculum of infection from 10^4^ cells/larvae to 10^7^ cells/larvae was analyzed. A reduction in survival over the 72 h incubation period was observed for all strains. The two LABs showed high survival, 55% for LAB1 and 75% for LAB 2 (Figure 6B,C), while the Y1 showed a survival of up to 40% after 72 h at the highest concentration tested. It is important to highlight that *P. kudriavzevii* strains were able to significantly reduce *G. mellonella* survival during the first 24 h (*p* < 0.0001). Specifically, after 24 h, a 30% reduction of larval survival rate was detected, whereas after 48 h the larval survival rate dropped by 40 (Figure 6A). In Figure 7, toxicity is reported of CFSs of single microorganisms and the CFS derived from the co-culture of all three together. All the CFSs were not toxic to the larvae until 72 h. In Table 6, cell density obtained by burden assay for *P. kudriavzevii* is reported. It is clearly evident that no proliferation has been detected for all the tested doses after 24 h of incubation.

## 4. Discussion

Probiotics are viable microorganisms generally added to foods to improve the diet, thanks to their nutritional value and potential health benefits, so that nowadays there is an increasing demand for new probiotic products [47,48]. Nevertheless, with probiotics there are safety concerns. The literature contains conflicting results regarding the positive and negative impacts of probiotics on human health and disease [3,49].

Probiotics do not work in the same manner for all people. Some authors have outlined the controversial efficacy of probiotics in some specific cases, such as prevention of Clostridium difficile-associated diarrhea [50], and other gastrointestinal syndromes [51].

Considering the complexity of the probiotic effects across different individuals, they should be consumed considering the specificity of the probiotic strains, the sources, the levels of exposure, the production properties, disease states, and general nutritional condition [52].

Probiotic microorganisms used in foods must be safe and effective, non-pathogenic and non-toxic, sensitive to antibiotics and antifungals, with antimicrobial activity, resistant to low pH and bile salts, and capable of colonizing in digestive tract environments.

Kefir is generally a low-production-cost homemade probiotic drink, considered a natural remedy due to the richness of microorganisms and the presence of metabolites produced by them. Kefir grains, measuring between 1 and 4 cm diameter, are composed of a natural matrix of exopolysaccharides and proteins, in which lactic acid bacteria, yeasts and acetic acid bacteria coexist in symbiosis [20,53].

Artisanal kefir, which is quite popular in Greece and other Eastern European countries, is becoming common also in Western Europe thanks to its proven benefits. During artisanal dairy kefir production, milk is inoculated with kefir grains and incubated for 1 day at room temperature. People prepare their artisanal kefir by procuring kefir grains from family, friends, in typical shops or on the internet, and the traditional methods used, vary depending on the type of kefir grain, the quantity of kefir grains added to the milk to be fermented, the time and temperature of fermentation, as well as the hygienic conditions practiced during the entire process.

Until now, large scale industrial production of kefir has been scarce due to the limitation of the methodological standardization crucial to its physiological benefits and health-promoting properties. Therefore, the characterization of probiotics must be carried out through standard in vitro experiments.

In this context, the present study is of significant importance, as it identified and evaluated the potential of a homemade kefir-derived yeast and lactic acid bacteria as probiotic candidates.

As part of its evaluation, we first isolated one yeast and two phenotypically different LABs, subsequently molecularly identified as *P. kudriavzevii* (Y1), *Lactococcus lactis* subsp. *hordniae* (LAB 1) and *L. lactis* subsp. *lactis* (LAB 2).

The results of tolerance to low pH and bile salts of the three isolates indicate that these strains are resistant to the acid and bile that would be encountered in the gastrointestinal environment. These results are a prerequisite for probiotic organisms and are in accordance with those previously reported for potentially probiotic strains associated with food, even if the ability to survive is variable and related to different kinds of food. The studied yeast isolate showed significant viability (more than 90%) even at 3% of bile concentration, according to the research of Helmy et al., in which *P. kudriavzevii* isolated from a cheese had tolerance to a bile concentration of 2% [54]. Our results showed, at the same time, that Y1 is able to withstand acidic pH 3, remaining viable until 4 h. This result was similar to a study of Wulan et al. [55], which showed that a *P. kudriavzevii* strain isolated from cocoa fermentation had the ability to survive at low pH. Both *Lactococcus lactis* subsp. *hordniae* and *L. lactis* subsp. *lactis* demonstrated a high resistance against acidic pH 3, while a decline in bacterial viability was observed after exposure to bile salt at a concentration of 0.3% during the first 4 h. The acid and bile tolerance of LABs vary for different species and strains, as reported by other researchers [56]. The isolates were screened for their adhesion ability, a key factor that determines yeast’s beneficial effects, a complex mechanism with a multi-step process, and a desirable property for a potential probiotic, using different tests. *Pichia kudriavzevii* showed 65% hydrophobicity, 95% auto-aggregation after 20 h, a high adhesion ability to Caco-2 and HT-29 cells confirmed by a co-aggregation capacity towards enteric pathogens, and finally a moderate ability to form biofilm in vitro, demonstrating the capacity to colonize and persist in host tissue. Our findings on hydrophobicity, aggregation and the adhesion to intestinal cells of the two LABs, desirable characteristics of probiotic strains, which help bacteria colonize and stay on the surface of the host mucosa, i.e., possessing a biofilm formation capacity, are consistent with previous studies reporting similar activities in LAB strains isolated from various sources [57].

Co-aggregation is one of the defensive mechanisms exerted by probiotics to create a competitive micro-environment around the pathogen, impeding colonization [58]. In our study, we evaluated co-aggregation capabilities using pathogenic bacteria, *E. coli*, *L. monocytogenes* and four *Salmonella* spp. The results indicated that the propensity for co-aggregation is evident after 4 h of incubation, both for Y1 and the two LAB strains. The ability of CFS to show antimicrobial activity against pathogens, considered a critical factor in the selection of probiotics, was investigated against Gram-positive and Gram-negative pathogens, such as *E. coli*, *L. monocytogenes* and different *Salmonella* spp. isolates. Y1 showed no effect, so we can hypothesize that competition against pathogens acts through other mechanisms, such as production of factors that neutralize bacterial toxins, or modulation of the host cell signaling pathway in the pro-inflammatory response during bacterial infection, as reported earlier [59]. Conversely, a broad-spectrum antimicrobial activity was demonstrated for LAB1 and LAB2, even if the antimicrobial activity of strains can vary depending on the source of isolation, the specific strain, and the target pathogen, as reported in other studies [60,61,62,63].

In this study, isolates LAB1, LAB2 and Y1 showed good antioxidant activity, which represents an important health benefit for the host by mitigating the dangerous effects of free radicals in the body. Research aims to identify new sources of this type of molecule and our results confirm the possibility of using these isolates as a natural source of antioxidants that bring health benefits [64,65,66,67].

Safety characteristics are necessary to evaluate potential probiotics. In our study, we evaluated the in vitro and in vivo safety of our isolates. All the strains were non-hemolytic and sensitive to the clinical antimycotic and antibiotics agents tested, in agreement with other findings regarding the factors that make bacteria and yeasts good probiotic candidates [68], since they should not carry transmissible resistance genes. Indeed, an important requirement for probiotic strains is that they should be, at the same time, resistant to antimicrobials in order to be protected during therapy or preventive activity, but it is also necessary to avoid resistant strains, which can limit effectiveness for human use [69,70].

*G. mellonella* larvae, which have the advantages that they can be incubated at human body temperature and their inoculum can be administered directly into their bodies, have been used as a model to assess microbial virulence [71]. Tran et al. [72] first evaluated the in vivo safety of a potential probiotic yeast strain isolated from fermented food in *G. mellonella* and reported that dosages below 10^6^ CFU/larva had over 90% survival with yeast density reduced to 10^2^–10^3^ CFU/larva after 72 h post-injection. Larvae injected with our isolates Y1, LAB1, and LAB2 were monitored until 72 h, and were shown to be totally healthy at injection doses of 10^5^ and 10^6^ CFU/larva, as with larvae injected with CFSs collected from cultures of each of the isolates, as well as from the mixed culture. The burden assay, carried out only for *P. kudriavzevii,* showed no evidence of proliferation at all tested concentrations and a decrease in cell density, maybe due to immune system activation. From these results, we can confirm the safety as probiotic of the three isolates at dosages below 10^6^ CFU/larva, but further investigations need to be conducted.

Based on the results obtained in this study, it can be concluded that the strains isolated from homemade kefir (*P. kudriavzevii*, *L. lactis* subsp. *hordniae* and *L. lactis* subsp. *lactis)* have demonstrated promising probiotic potential and safety characteristics for use as a starter culture to produce probiotic-based food products. Further investigation is needed to validate their potential health benefits, such as their immunomodulatory and antiinflammatory properties, so that broader applications for human health can be found. In this way, the characterization of the new isolates can be addressed, in the perspective of broadening the field of potential probiotic strains.

## 5. Conclusions

Our study demonstrated that the three strains isolated from homemade kefir were able to tolerate and survive in simulated gastric and intestinal juices, and not only expressed remarkable cell surface characteristics such as hydrophobicity, auto-aggregation, and co-aggregation, but also had antioxidant power and biofilm formation ability. Our results also indicate that these strains were able to adhere to intestinal cell lines, reducing the colonization and invasion of some intestinal pathogenic bacteria, and are safe, as shown by in vitro and in vivo tests. For these interesting probiotic properties, they could be considered promising candidates for biotechnological future developments.

## Figures and Tables

**Table 1:** Results of Sanger Sequencing performed on bacterial and fungal colonies isolated from the samples under analysis. Obtained FASTA Sequences were compared to NCBI Sequence Database sequences, using BLASTN ver. 2.2.29.

Internal Code	Identified Microorganism	Max Score	Total Score	Query Cover	e-Value	% Identity	Accession N.
LAB1	*Lactococcus lactis* subsp. *hordniae* strain V4048	1256	1256	98%	0.0	99.57%	OR755425.1
LAB2	*Lactococcus lactis* subsp. lactis strain NM26-6	1286	1286	99%	0.0	99.72%	HM218132.1
Y1	*Pichia kudriavzevii* strain CBS 5147	1037	1037	99%	0.0	99.65%	MH545928.1

**Table 2:** Percentage of co-aggregation of Y1, LAB1 and LAB2 with pathogenic bacteria after 2 h and 4 h incubation at 37 °C.

	*E. coli* ATCC 25922		*L. monocytogenes* ATCC 7644		*Salmonella* spp. S1		*Salmonella* spp. S2		*Salmonella* spp. S3		*Salmonella* spp. S4	
Time (h)	2	4	2	4	2	4	2	4	2	4	2	4
Y1	36.3 ^a^ ± 1.4	79.1 ^b^ ± 1.4	43.3 ^a^ ± 2.1	81.6 ^b^ ± 1.3	10.9 ^c^ ± 1.1	68.2 ^b^ ± 2.3	14.6 ^c^ ± 0.4	51.9 ^d^ ± 3.2	32.1 ^a^ ± 1.6	82.6 ^b^ ± 2.7	40.3 ^a^ ± 1.6	79.8 ^b^ ± 2.5
LAB1	8.0 ^a^ ± 1.6	25.3 ^b^ ± 2.0	7.0 ^a^ ± 0.5	31.3 ^c^ ± 2.5	6.2 ^a^ ± 0.3	34.1 ^c^ ± 1.4	10.2 ^a^ ± 2.3	44.0 ^d^ ± 1.5	18.1 ^e^ ± 0.5	30.0 ^c^ ± 1.0	7.6 ^a^ ± 0.2	21.3 ^e^ ± 1.5
LAB2	9.0 ^a^ ± 1.1	22.5 ^b^ ± 2.1	6.4 ^a^ ± 0.9	24.9 ^b^ ± 2.3	17.2 ^c^ ± 1.4	29.6 ^b^ ± 1.9	21.0 ^b^ ± 2.8	51.2 ^e^ ± 3.1	15.1 ^c^ ± 1.5	28.3 ^b^ ± 0.3	9.7 ^a^ ± 1.4	20.7 ^b^ ± 2.8

^a, b, c, d, e^ Significantly different (*p* < 0.05) in the same row.

**Table 3:** Biofilm formation on polystyrene plates by the different isolates in single or mixed-culture. Results were obtained after 24 h of incubation using crystal violet (CV) staining and CFU plates’ count.

	OD_570_	CFU/mL		Classification
		Y	LAB	
Y1	0.246	1.7 × 10^4^	-	moderate
LAB1	0.148	-	4.0 × 10^3^	weak
LAB2	0.159	-	1.4 × 10^3^	weak
Y1 + LAB1	0.299	3.1 × 10^4^	8.1 × 10^3^	moderate
Y1 + LAB2	0.231	2.0 × 10^4^	6.5 × 10^3^	moderate
Y1 + LAB1 + LAB2	0.397	2.1 × 10^4^	3.3 × 10^4^	moderate

Y = yeast strain; LAB = lactic acid bacteria.

**Table 4:** Antimicrobial activity against food-borne pathogens. Diameter (Ø mm) of inhibition zone of CFS of Y1, LAB 1 and LAB 2.

	CFS-Y1	CFS-LAB1	CFS-LAB2	CFS-MIX
	Ø mm			
*E. coli* ATCC 25922	-	-	-	-
*L. monocytogenes* ATCC 7644	-	11.5 ^a^	12.6 ^a^	11.3 ^a^
*Salmonella* spp. S1	-	8.2 ^b^	8.0 ^b^	10.2 ^a^
*Salmonella* spp. S2	-	-	-	-
*Salmonella* spp. S3	-	7.1 ^b^	7.9 ^b^	8.7 ^b^
*Salmonella* spp. S4	-	11.0 ^a^	11.9 ^a^	12.2 ^a^

^a, b^ Significantly different (*p* < 0.05).

**Table 5:** Antimicrobial resistance assay of Y1, LAB 1 and LAB 2. The strains were considered susceptible (S), intermediate (I) or resistant (R).

	GM	AMP	PB	AMC	NOR	STR	CTX	CIP	AZ	CRO	VAN	RIF	FLC	CSF	KET	ITR	AmpB
Y1	R	R	R	R	R	R	R	R	R	R	R	R	S	S	S	R	S
Lab1	S	S	S	S	S	S	S	S	S	S	S	S	-	-	-	-	-
Lab2	S	S	S	S	S	S	S	S	S	S	S	S	-	-	-	-	-

**Table 6:** Density of *P. kudriavzevii* in *G. mellonella* larvae after 24 h of incubation.

Injection Doses	Yeast Density (CFU/mL)
1 × 10^4^	1.3 ± 0.45 × 10^3 a^
1 × 10^5^	1.4 ± 0.38 × 10^3 a^
1 × 10^6^	6.5 ± 0.06 × 10^3 a^
1 × 10^7^	7.9 ± 0.25 × 10^5 b^

^a, b^ Significantly different (*p* < 0.05).

## Data Availability

The original contributions presented in the study are included in the article, further inquiries can be directed to the corresponding author.
